# Elevated CDC45 Expression Predicts Poorer Overall Survival Prognoses and Worse Immune Responses for Kidney Renal Clear Cell Carcinoma via Single-Cell and Bulk RNA-Sequencing

**DOI:** 10.1007/s10528-023-10500-y

**Published:** 2023-08-29

**Authors:** Xinyu Zhang, Jianhua Zhou, Yong Wang, Xing Wang, Bingye Zhu, Qianwei Xing

**Affiliations:** 1 Department of Urology, Affiliated Hospital of Nantong University, No.20 West Temple Road, Nantong, 226001 Jiangsu Province China; 2 Department of Urology, Affiliated Nantong Hospital of Shanghai University (The Sixth People’s Hospital of Nantong), No. 881 Yonghe Road, Nantong, 226001 Jiangsu Province China; 3 Department of Urology, Shanghai Jiangqiao Hospital, Shanghai General Hospital Jiading Branch, Jiading District, Shanghai, 201803 China; 4 Department of Urology, Lanzhou University Second Hospital, Lanzhou, 730000 Gansu Province China; 5 Department of Urology, Zhenjiang Hospital of Chinese Traditional and Western Medicine, Zhenjiang, 212000 Jiangsu Province China

**Keywords:** Kidney renal clear cell carcinoma, Overall survival, CDC45, Immunity, TCGA

## Abstract

The main objective of this paper is to analyze the prognostic and immunological value of CDC45 in kidney renal clear cell carcinoma (KIRC) using single-cell and bulk RNA-sequencing approaches. The expression of CDC45 in KIRC was evaluated by the HPA database, the TCGA-KIRC dataset and verified by PCR analysis and single-cell RNA-sequencing. The ability of CDC45 to independently predict prognosis in KIRC was confirmed by univariate/multivariate regression analysis. Gene set enrichment analysis (GSEA) was employed to explore CDC45-related pathways in KIRC. In addition, Relationships between CDC45 and immunity were also examined. Elevated CDC45 expression in KIRC was demonstrated at mRNA and protein levels. The results of the correlation analysis showed that as CDC45 expression increased, so did the histological grade, clinical stage, and TNM stage of the patients (*p* < 0.05). Univariate/multivariate regression analysis suggested CDC45 as an independent prognostic factor for KIRC. Seven pathways related to CDC45 were screened through GSEA. Meanwhile, we found that CDC45 was correlated with tumor mutational burden (TMB) and microsatellite instability (MSI) but not tumor neoantigen burden (TNB). Regarding immunity, CDC45 exhibited correlations with the tumor microenvironment, immune cell infiltration, and immune checkpoints. Besides, low CDC45 expression was shown to be associated with a better response to immunotherapy. Single-cell RNA-sequencing revealed that CDC45 was differently expressed in T cells (*p* < 0.05). CDC45 showed potential as a prognostic biomarker and therapeutic target for KIRC. Meanwhile, the CDC45 low expression group was more sensitive to immunotherapy.

**Supplementary Information:**

The online version contains supplementary material available at 10.1007/s10528-023-10500-y.

## Introduction

In urology, renal cell carcinoma (RCC) was a common form of cancer, second only to bladder cancer in terms of incidence. Mortality from RCC was rising, with an increasing annual incidence globally (Hsieh et al. 2017). Kidney renal clear cell carcinoma (KIRC), the most common type, accounted for 70–85% of cases (Escudier et al. 2019). With the improvement of medical technology and the popularization of medical checkups, the early diagnosis rate of KIRC had improved. Despite this, in 30% of cases, cancer was still in an advanced stage. The prognosis for advanced KIRC was poor, with a 5-year survival rate as low as 11.7% (Siegel et al. 2017). Research studies had shown that the incidence of postoperative metastases in KIRC patients was 30% or more, and neither postoperative nor preoperative metastases had a very good survival rate. Metastatic KIRC featured a 5-year survival rate of only 10% (Wang et al. 2018). As such, the search for novel biomarkers and therapeutic targets was crucial to improving KIRC patient outcomes.

It had been reported that cell-division cycle proteins (CDC) were commonly found in cells that were actively in the process of division, but were downregulated during cell quiescence, differentiation, or senescence (Kingsbury et al. 2005). CDC45 was originally discovered during genetic screening for yeast cell cycle mutants (Hennessy et al. 1991). Later studies revealed that CDC45 was one of the essential factors in the process of DNA replication (Hardy 1997; Zou et al. 1997). Since then, numerous studies had demonstrated that CDC45 was necessary for the entire process of DNA replication, including the establishment of the initiation complex, the unhealing of chromosomes at the replication fork, and the orderly synthesis of DNA (Pacek and Walter 2004). In the field of oncology, CDC45 had also been extensively studied. It had been reported that CDC45 was commonly highly expressed in various malignant tumors such as breast cancer, cervical cancer, lung cancer, and glioblastoma (Tomita et al. 2011), and that CDC45 may be a marker for the proliferation of various malignant tumors (Pollok et al. 2007). However, the research on the mechanism of CDC45 in the field of KIRC is not thorough. Recently, the immune microenvironment in cancer patients has received extensive attention. Its complexity and diversity have affected the occurrence and development of cancer, providing a new idea for clinical immunotherapy. Therefore, here we examined the prognosis of KIRC in relation to CDC45 and further investigated the associations between CDC45 and immunity and immunotherapy.

## Materials and Methods

### Data Collection

The Cancer Genome Atlas (TCGA; http://cancergenome.nih.gov/) provided the CDC45 expression matrix and clinical data for 539 KIRC and 72 normal kidney samples. Following this, incomplete or missing data were eliminated, and the CDC45 gene expression profile and corresponding clinical information were used for further analysis. All Analyses were conducted using R software (https://www.r-project.org/). The different expression levels of CDC45 mRNA were conducted by the R package "limma". In addition, we took |log2 fold change (FC)|≥ 1, and adjusted p-value < 0.05 as the definition of cut-off criteria.

### Detection of CDC45 Expression in KIRC and Enrichment of Functions

The “limma” was applied to detect differently expressed genes (DEGs) between tumors and adjacent normal tissues, including the CDC45 gene in KIRC. CDC45 expression at the protein level was verified by immunohistochemical methods applying the human protein atlas (HPA, http://www.proteinatlas.org/).

### Collection of Clinical Samples and Quantitative Real‑Time PCR (qRT‑PCR)

To increase the credibility of the data, we collected eight pairs of tissues from patients with KIRC who underwent nephrectomy at the Affiliated Hospital of Nantong University to further validate CDC45 expression at the mRNA level. The details of these eight patients were as follows: age 50 to 80 years; weight 55 to 85 kg; three males and five females; T stage T1-T4; N stage N0-N1; and M stage all M0. RNA was extracted from freshly frozen KIRC samples using the TRIzol reagent (Life Technology, USA). In accordance with the instructions, reverse transcription was performed on RNA. Finally, CDC45 expression was detected with the help of the SYBR Green reagent (Vazyme, Nanjing, China). GAPDH served as a standardized control for KIRC specimens. Relative CDC45 expression was calculated using the 2^−ΔΔCt^ method. The above data analysis was based on GraphPad Prism 8.0.1 and a t-test. The corresponding primers in this study were: CDC45 (F: 5’-TGAGTATGACCTCCGCCTGG-3’, R: 5’-CCATGCACAGACCACAGCTT-3’) and GAPDH (F: 5’-CAGGAGGCATTGCTGATGAT-3’, R: 5’-GAAGGCTGGGGCTCATTT-3’).

### Univariate/Multivariate Cox Hazard Regression Analysis

To further investigate the effect of CDC45 on overall survival (OS), we used the R package to perform univariate/multivariate regression analysis on KIRC patients in the TCGA database to determine whether CDC45 was an independent factor associated with OS by eight clinical factors (race, sex, age, grading, T, N, M, stage). And we presented it in the form of a forest plot using the R language.

### Gene Set Enrichment Analysis (GSEA)

The TCGA-KIRC samples were stratified into high- and low-CDC45 groups. Following this, the potential mechanism of CDC45 in KIRC was explored by GSEA where *p* < 0.05 and FDR *q* value < 0.25 were regarded as the cut-off criteria (Hänzelmann et al. 2013).

### Correlation Analysis of CDC45 with Microsatellite Instability (MSI), Tumor Mutational Burden (TMB) and Tumor Neoantigen Burden (TNB)

To further analyze the possible key genes in KIRC, we constructed and visualized PPI networks using the STRING database and Cytoscape software. Moreover, to examine the association between CDC45 expression and MSI, TMB, and TNB, we took advantage of the Sangerbox website (http://www.sange rbox.com/tool), while setting the threshold to less than 0.05 (Cai et al. 2020; Li et al. 2021; Liu et al. 2021a).

### Immunotherapy Response Prediction and Correlations between CDC45 and Immunity

Based on the ESTIMATE algorithm and CDC45 expression matrix, the ImmuneScore, StromalScore, and ESTIMATEScore were calculated (Yu et al. 2022). TIMER was employed to examine the relationships between CDC45 and immune cell infiltration in KIRC (TIMER, https://cistrome.shinyapps.io/timer/) (Chen et al. 2021). Correlation analysis examined the CDC45-immune checkpoint and cell associations. These correlation analyses were done with the help of the Sangerbox tool. Immunotherapy outcomes were calculated by uploading the tumor expression matrix to the TIDE (tumor immune dysfunction and exclusion) database (http://tide.dfci.harvard.edu) and TIGER (Tumor Immunotherapy Gene Expression Resource) database (http://tiger.canceromics.org/) (Chen et al. 2022; Jiang et al. 2018).

### Detection of CDC45 Expression in Various Cells in the Tumor Microenvironment by Single-Cell Sequencing

The TISCH (http://tisch.comp-genomics.org) database integrated single-cell sequencing data for 27 cancers, providing gene expression visualization at the single-cell level (Sun et al. 2021). This study used the TISCH database to explore the single-cell level expression of CDC45 in KIRC in the GSE159115 dataset.

## Results

### Expression and External Verification of CDC45 in KIRC

**Fig. 1:**
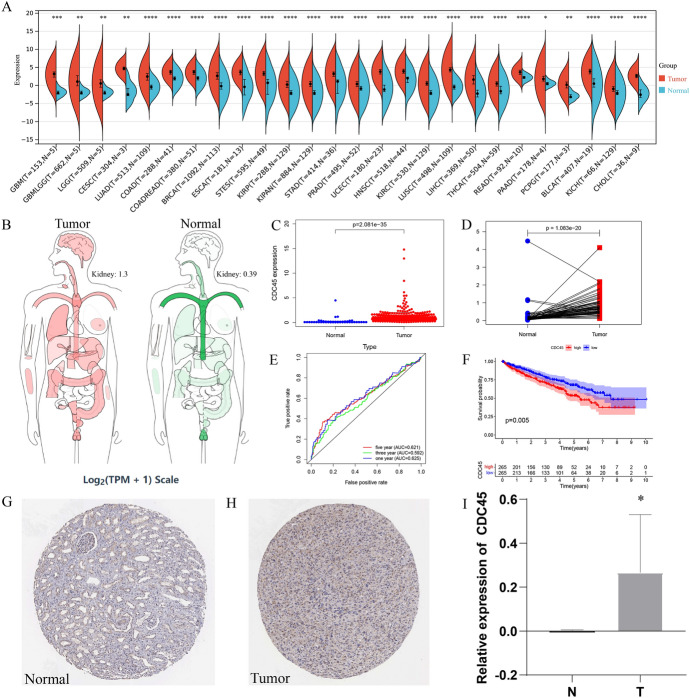
The expression of CDC45 in KIRC. **A** Expression of CDC45 in various cancers in TCGA database; **B** Expression and distribution of genes in human body; **C** Boxplot of CDC45 expression in normal renal tissue and KIRC tissue; **D** Pairwise boxplot of CDC45 expression in normal renal tissue and KIRC tissue; **E** ROC curve of CDC45 and its AUCs of 1-, 3-, and 5-year survival. **F** K–M survival analysis of CDC45. **G**–**H** Immunohistochemical staining of CDC45 in HPA database. **I** Verification of CDC45 expression at tissue level by PCR, T represented KIRC tissue; N represented adjacent normal tissue. * *p* < 0.05, ** *p* < 0.01, *** *p* < 0.001

To investigate the expression of CDC45 in tumors and adjacent normal tissues, the expression levels of CDC45 were examined in pan-cancers (Fig. 1A). Meanwhile, we could see that CDC45 expression had a significant increase in tumor tissues than in normal tissues (*p* < 0.05, Fig. 1B–C). Pairwise boxplot also suggested the same results (*p* < 0.05, Fig. 1D). One, three, five-year area under the curve (AUC) values of CDC45 in KIRC of OS prognosis were 0.625, 0.592 and 0.621(Fig. 1E). By K-M survival analysis we concluded that elevated CDC45 expression predicted poor OS (Fig. 1F). Immunohistochemical staining of CDC45 downloaded from the HPA database (https: //www.proteinatlas.org/) also clearly revealed that normal tissues had a lower expression of CDC45 than KIRC (Fig. 1G–H). In addition, validation results of PCR at the mRNA level further indicated that CDC45 expression was elevated in KIRC (Fig. 1I).

### Relationships between CDC45 and Clinicopathologic Features

**Fig. 2:**
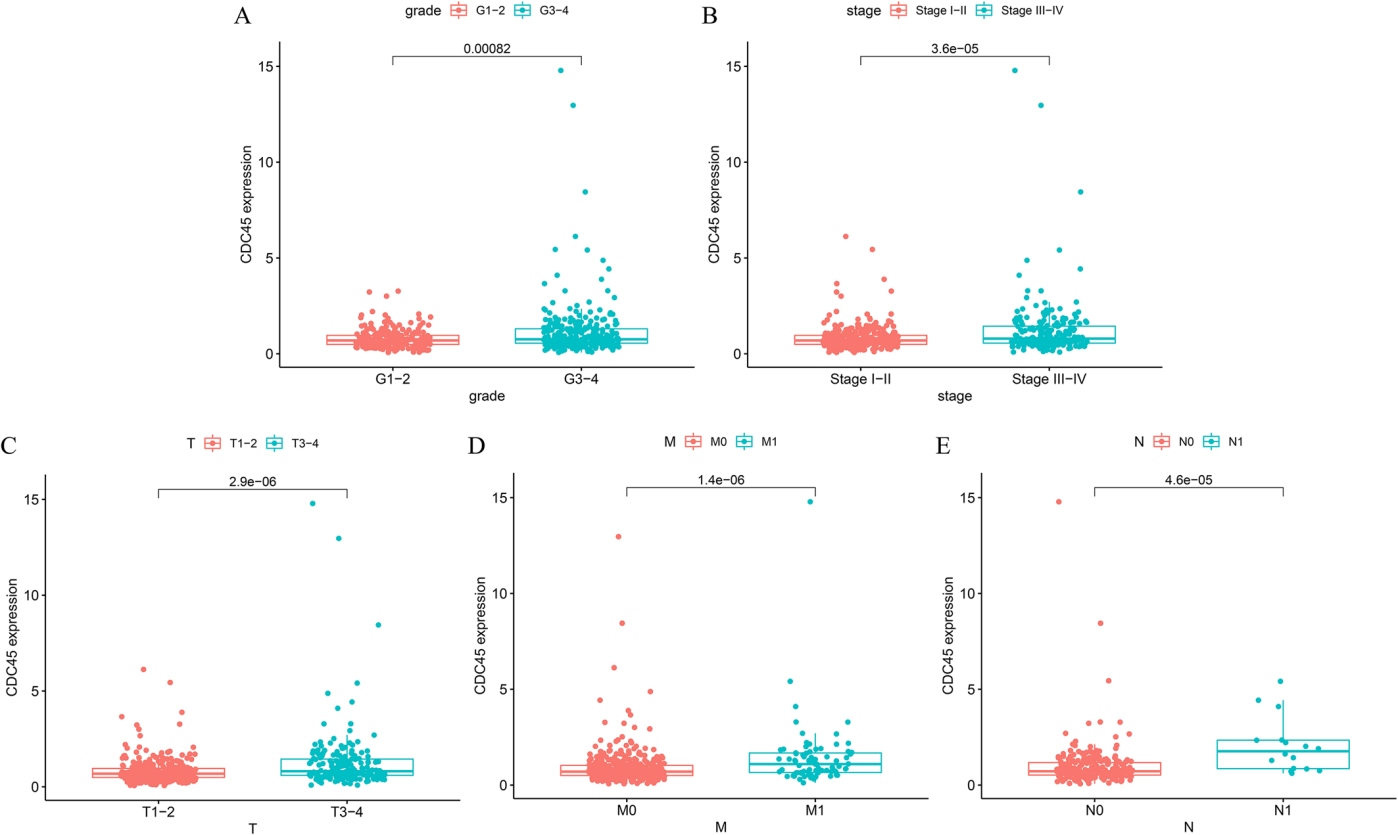
Associations between CDC45 and **A** grade, **B** stage **C** T, **D** M, **E** N

Logistic analysis was used to screen the clinicopathologic data of TCGA-KIRC patients to determine the relationships between five clinical features and CDC45. Elevated CDC45 expression was significantly correlated with higher grade (*p* = 0.00082), stage (*p* = 3.6E-05), T (*p* = 2.9E-06), M (*p* = 1.4E-06) and N (*p* = 4.6E-05) (Fig. 2). Considering the above findings, we speculated that elevated CDC45 expression was associated with the progression of KIRC.

### Prognostic Analysis of CDC45 in KIRC

**Fig. 3:**
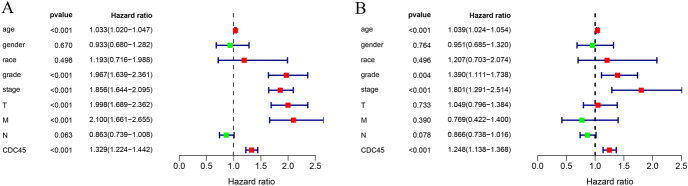
Validation of whether CDC45 can be used as an independent prognostic factor for KIRC. **A** Univariate Cox regression analyses; **B** multivariate Cox regression analyses

Univariate regression analysis reflected that there was a significant association between OS and age, grade, stage, T, M, CDC45 expression (all *p* < 0.05; Fig. 3A and Table 1). Multivariate regression analysis reflected that age, grade, stage, and CDC45 expression of KIRC were correlated with OS in KIRC (all *p* < 0.05; Fig. 3B and Table 1). The p values of CDC45 in the above two analyses were significant, which further indicated that CDC45 may be an independent prognostic factor for KIRC.

**Table 1:** Univariate and multivariate analyses of CDC45 and clinicopathologic factors of overall survival in KIRC

Factors	Univariate analysis				Multivariate analysis			
	HR	HR.95L	HR.95H	*p *value	HR	HR.95L	HR.95H	*p* value
Age	1.033274	1.019678	1.047052	1.28E-06	1.03896	1.023814	1.054331	3.38E-07
Gender	0.933298	0.679692	1.28153	0.669603	0.951029	0.685278	1.319839	0.763958
Race	1.193075	0.71596	1.988138	0.498059	1.207062	0.702563	2.073834	0.495543
Grade	1.966884	1.638836	2.360598	3.70E-13	1.389709	1.111427	1.737668	0.003894
Stage	1.855626	1.643637	2.094956	1.71E-23	1.80147	1.290699	2.514369	0.00054
T	1.997582	1.689052	2.362469	6.29E-16	1.049274	0.795576	1.383875	0.73341
M	2.099647	1.660681	2.654644	5.70E-10	0.769068	0.422445	1.400101	0.390344
N	0.862971	0.73887	1.007916	0.062826	0.866246	0.738363	1.016279	0.078098
CDC45	1.328505	1.223968	1.441971	1.10E-11	1.247769	1.137991	1.368138	2.46E-06

### Identification of CDC45-Related Signaling Pathways

**Fig. 4:**
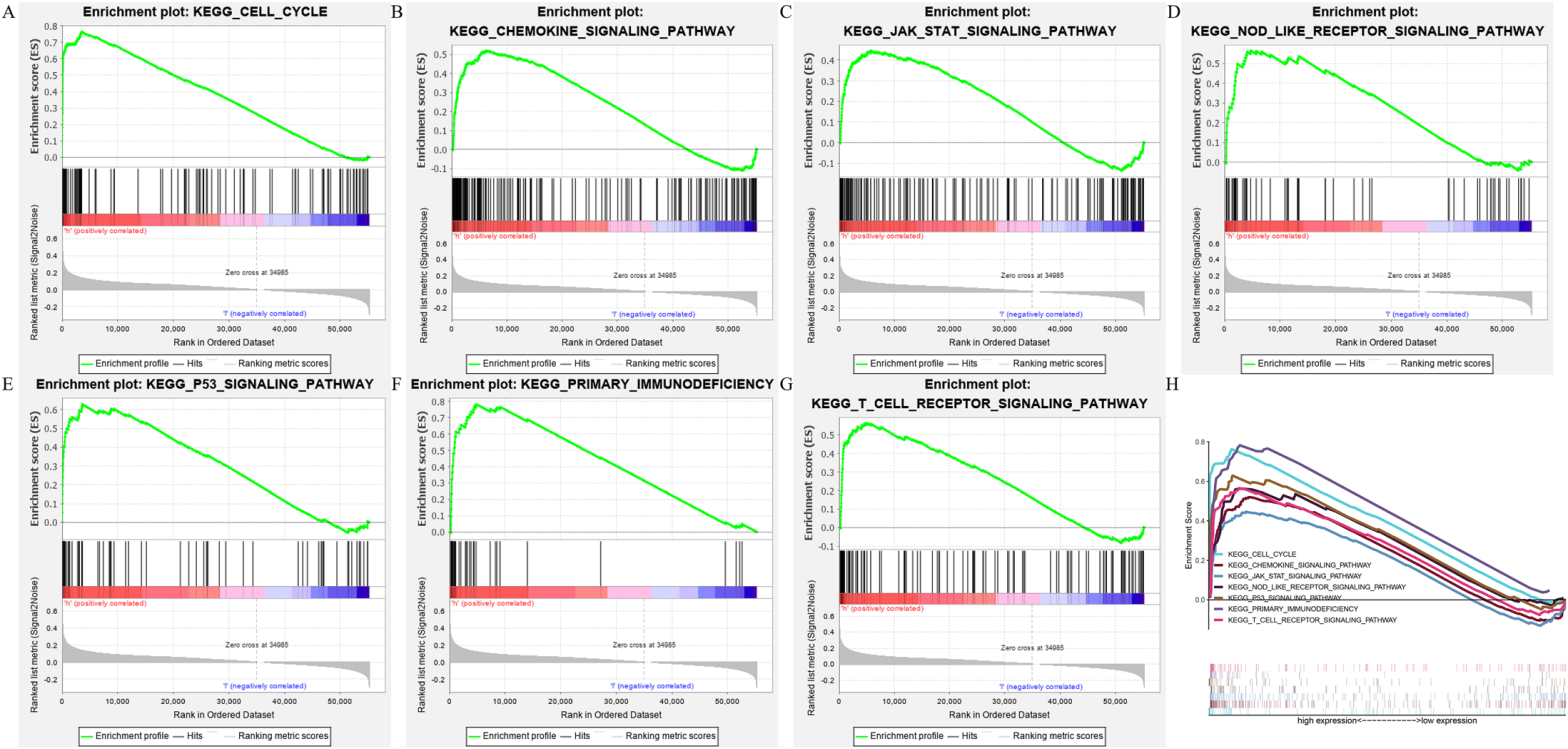
Enrichment plots from gene set enrichment analysis (GSEA). **A** CELL CYCLE; **B** CHEMOKINE signaling pathway; **C** JAK STAT signaling pathway; **D** NOD LIKE RECEPTOR signaling pathway; **E** p53 signaling pathway; **F** PRIMARY IMMUNODEFICIENCY; **G** T CELL RECEPTOR signaling pathway; **H** seven CDC45-related signaling pathways

To further explore pathways associated with CDC45, GSEA assays were taken between high- and low-CDC45 matrices, and seven signaling pathways, including CELL CYCLE, CHEMOKINE, JAK STAT, NOD LIKE RECEPTOR, p53, T CELL RECEPTOR SIGNALING PATHWAY, PRIMARY IMMUNODEFICIENCY were identified by setting the *p* < 0.05 and FDR *q* value < 0.25 (Fig. 4 and Table 2). These findings might provide insights into CDC45-mediated KIRC pathogenesis.

**Table 2:** Gene set enrichment analysis (GSEA) of CDC45 in KIRC

GeneSet name	NES	Nominal *p* value	FDR *q* value
Cell_cycle	2.556	0.000	0.000
Chemokine_signaling_pathway	1.976	0.014	0.018
JAK_STAT_Signaling_pathway	1.864	0.014	0.032
NOD_like_receptor_signaling_pathway	1.960	0.008	0.019
P53_signaling_pathway	2.258	0.000	0.002
Primary_immunodeficiency	2.128	0.000	0.006
T_cell_receptor_signaling_pathway	1.976	0.014	0.018

### Associations between CDC45 and PPI, TMB, TNB, MSI in KIRC

**Fig. 5:**
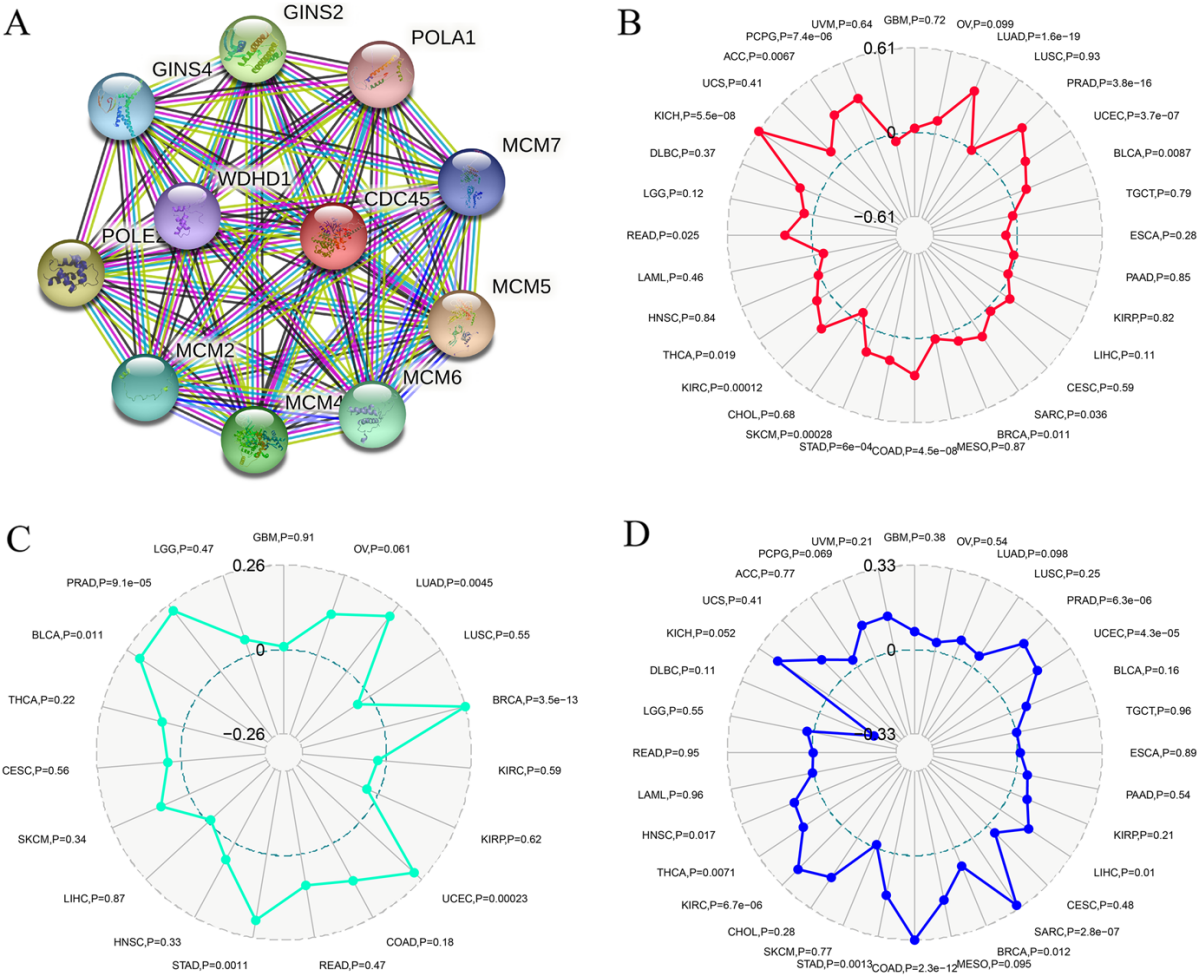
Relationships between CDC45 and **A** PPI; **B** TMB; **C** TNB; **D** MSI

We constructed a PPI network through the String database (https://string-db.org/) to search for genes in KIRC that may be closely related to CDC45 (Szklarczyk et al. 2011). A total of ten genes closely related to CDC45 were finally obtained (GINS2, GINS4, WDHD1, POLE2, MCM2, MCM4, MCM6, MCM5, MCM7, POLA1, Fig. 5A). Meanwhile, we evaluated the relationships between CDC45 and MSI, TMB, TNB through the Sangerbox website with a threshold of p < 0.05. According to the findings, CDC45 was significantly connected with MSI as well as TMB but not TNB in KIRC (Fig. 5B–D).

### Association of CDC45 with KIRC Tumor Microenvironment, Tumor Immune Infiltration, Immune Cell Pathways, and Immune Checkpoint

**Fig. 6:**
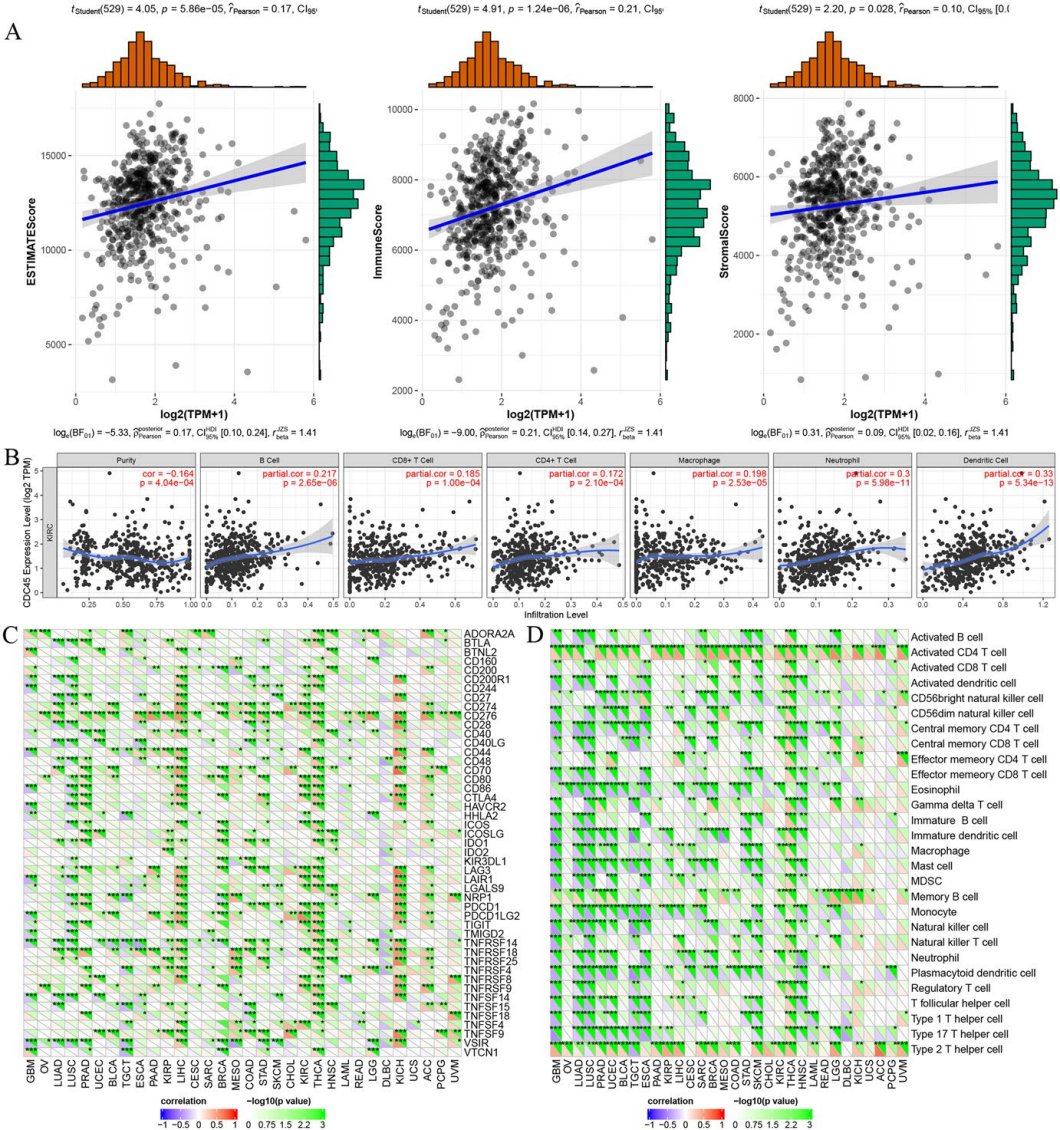
Relationships between CDC45 and **A** tumor microenvironment; **B** immune infiltrations; **C** immune checkpoint molecules; **D** immune cells. *p < 0.05, **p < 0.01, ***p < 0.001

Based on the results of the correlation analysis between CDC45 and immunity, we found that CDC45 had a strong link to ImmuneScore, ESTIMATEScore, and StromalScore (*p* < 0.05, Fig. 6A). In terms of tumor immune infiltration, CDC45 was significantly associated with B cell, CD8 + T cell, CD4 + T cell, neutrophil, dendritic and macrophage cell infiltration (all *p* < 0.001, Fig. 6B). Meanwhile, the data from TCGA showed that CDC45 was associated with immune checkpoints such as PDCD1, PDCD1LG2, TNFSF4, in KIRC (all *p* < 0.05; Fig. 6C). In a co-expression analysis, CDC45 was significantly correlated with immune cell pathways like activated CD4 T cell, central memory CD8 T cell, monocyte, etc. (all *p* < 0.05, Fig. 6D). The above results concluded that CDC45 was significantly associated with immunity in four aspects.

### CDC45-Related Immunotherapy Response Prediction

**Fig. 7:**
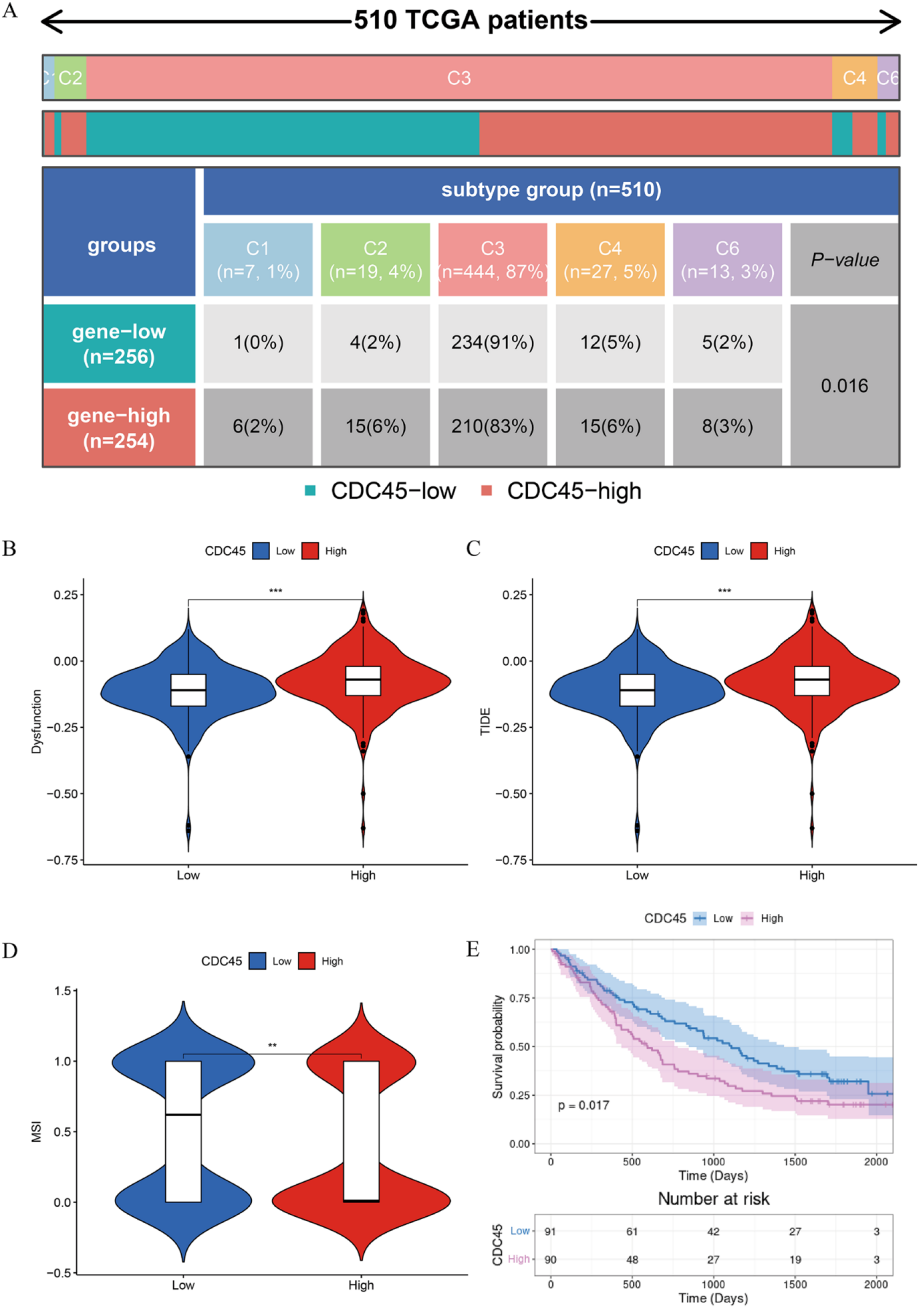
Prediction of CDC45-related immune responses of immunotherapy. **A** Proportion of high and low expression groups of CDC45 in immune subtypes. **B** Differences of CDC45 expression in T cell dysfunction scores; **C** Differences of CDC45 expression in TIDE scores; **D** Differences of CDC45 expression in MSI; **E** Survival analysis between high and low CDC45 expression groups. **p < 0.01, ***p < 0.001

Based on our results, CDC45 was differentially expressed across immune subtypes, including C1, C2, C3, C4, and C6 (Thorsson et al. 2018) (Fig. 7A). Among them, CDC45 accounted for the highest proportion in the C3 immune subtype. According to Fig. 7B–D, KIRC patients with high CDC45 expression had lower MSI scores, higher tumor immune dysfunction scores and higher TIDE scores. Accordingly, these patients may have a worse outcome following immunotherapy than those with low CDC45 expression. Meanwhile, we further validated the impact of the relationships between CDC45 and immunotherapy on the prognosis of KIRC through TIGER. It could be seen from Fig. 7E that the prognosis was worse for patients whose levels of CDC45 were high. The whole figure reflected that elevated CDC45 expression was associated with a poorer immune response; thus those patients with elevated CDC45 expression who were not sensitive to immunotherapy naturally had a worse prognosis.

### Results of Single-Cell Sequencing

**Fig. 8:**
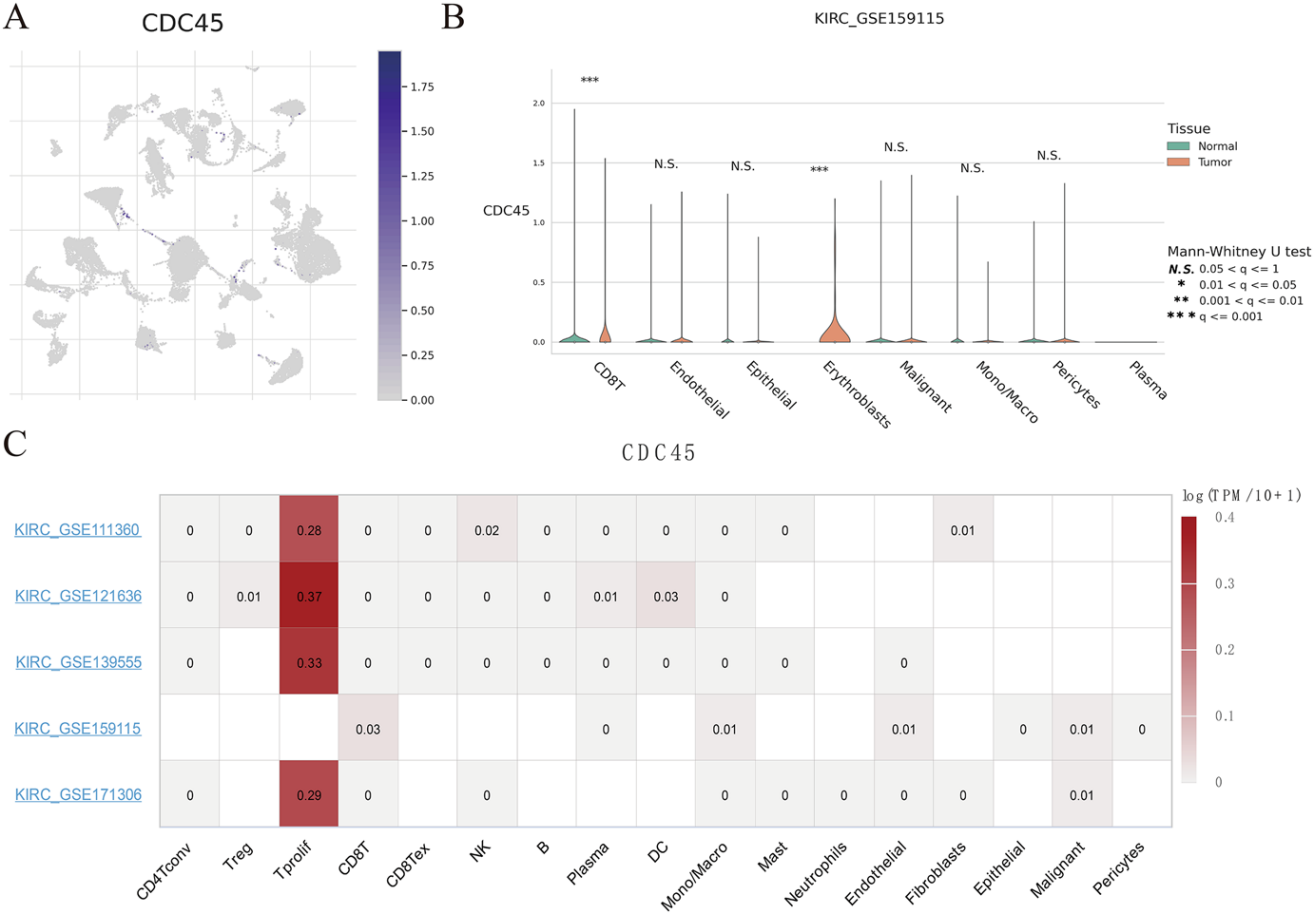
CDC45 expression in various cells in the tumor microenvironment by single-cell sequencing. **A** UMAP plot CDC45 in various cells in the GSE159115 dataset. **B** Violin plot of CDC45 expression in immune cells and stromal cells of KIRC tissue and normal tissue in the GSE159115 dataset. **C** The results of the expression of CDC45 in various cells in five datasets. *p < 0.05, **p < 0.01, ***p < 0.001

In parallel, we used the TISCH dataset to further validate the expression of CDC45 in the tumor microenvironment of KIRC at the single-cell level. In GSE159115, CDC45 was mainly expressed in CD8 T, Mono/Macro, endothelial, and malignant cells (Figure S1; Fig. 8A). The GSE159115 violin plot suggested that the expression of CDC45 in KIRC CD8 T cells as well as Erythroblasts was significantly different from that of CDC45 in corresponding normal tissue CD8 T cells as well as Erythroblasts (Fig. 8B). In addition, the results of CDC45 in four additional datasets showed significant expression of CDC45 in Tprolif (Fig. 8C). The results of single-cell sequencing showed that CDC45 was mainly expressed in T cells, partially explaining its immune correlations.

## Discussion

KIRC was a highly heterogeneous urological malignancy, and the biological mechanism of its development was not fully understood. Targeted therapy was the main treatment modality for patients with advanced KIRC, but studies had shown that only 20–40% of patients benefited from it, and most high-risk patients still had a poor prognosis. The search for biomarkers with high specificity and sensitivity was important for targeted individualized therapeutic treatment and prediction of the prognosis of patients with KIRC (Barata and Rini 2017; Li et al. 2019a). Therefore, this study explored the possibility of CDC45 as a potential therapeutic target for KIRC based on bioinformatics.

The results of the paper reflected that CDC45 was highly expressed in a variety of human malignancies and was also upregulated in KIRC tissues. We also verified the expression level of CDC45 in KIRC using HPA and PCR. Clinicopathological correlation analysis showed that as CDC45 expression increased, so did the histological grade, clinical stage, and TNM stage of the patients (*p* < 0.05). Univariate/multivariate analyses suggested CDC45 as an independent prognostic factor associated with OS in KIRC. Based on the above results, the paper hypothesized that CDC45 may be a potential oncogenic factor of KIRC and was expected to be a potential prognostic biomarker of KIRC. Seven signaling pathways related to CDC45 were obtained by applying GSEA, including CELL CYCLE, CHEMOKINE, JAK STAT, NOD LIKE RECEPTOR, P53, PRIMARY IMMUNODEFICIENCY, T CELL RECEPTOR signaling pathways. In addition, we analyzed and concluded that CDC45 was closely related to the tumor microenvironment, immune cell infiltration, and immune checkpoint. It was further concluded by TIDE that low CDC45 expression was associated with better immunotherapy efficacy.

CDC45 had been considered to be an indispensable factor in DNA replication since its discovery. DNA replication was a critical step in the mid-cell phase and required tight control to ensure the accuracy of genomic information. Abnormal replication could lead to genetic instability and cancer (Hills and Diffley 2014). As the study of CDC45 progresses, more and more researchers had explored the expression of CDC45 in tumor cells. A high level of expression of CDC45 was found in different types of tumor cells, and in the study of the mechanism, it was found that CDC45 mainly regulated the abnormal expression of cyclin through cell cycle, which caused tumorigenesis (Hanahan and Weinberg 2000). Sun et al. showed that elevated CDC45 expression was strongly correlated with tumor size and grade, and demonstrated that silencing of CDC45 inhibited tumor growth through cell cycle G1 phase arrest and induction of apoptosis by gene silencing in papillary thyroid cancer (Sun et al. 2017). Li et al. showed that CDC45 was higher in malignant squamous cell carcinoma than in mild precancerous lesions, and the expression level tended to increase with the grade of precancerous lesions from mild, moderate to high (Li et al. 2008). In the exploration of large sample data in the TCGA database, CDC45 was found to be a key tumor-associated gene in cervical cancer (Zhang and Zhao 2016), prostate cancer (Li et al. 2017) and lung cancer (Zhang et al. 2014), and the analysis of prognostic correlation showed that CDC45 was significantly related to poor prognosis in non-small cell lung cancer (Piao et al. 2018) and pancreatic cancer (Haider et al. 2014). In conclusion, tumors were developed in part by CDC45.

To provide insights into potential CDC45-mediated KIRC pathogenesis, we identified seven corresponding pathways through GSEA. Some of these pathways had been extensively studied in the field of oncology. P53 signaling was an important intracellular oncogenic pathway. Lacroix et al. showed that P53 and P21 in this pathway were biologically active after phosphorylation and widely affected the physiopathological processes of tumor cell metabolism, proliferation, cell cycle, and metastasis and invasion (Lacroix et al. 2020). Vennin et al. showed that P53 signaling inhibited epithelial mesenchymal transition in pancreatic cancer cells by regulating transcription of downstream related genes and driving phosphorylation of related oncogenic molecules such as P21 (Vennin et al. 2019). Wang et al. showed that in prostate cancer, CREB1 regulated P53 activity and inhibited angiogenesis in tumor cells (Wang et al. 2019). JAK/STAT pathway was an important pathway for cytokine signaling and was of great interest in tumors. Studies had shown that inhibiting of the IL-6/JAK2/STAT3 signaling pathway inhibited the proliferation of glioma cells and promotes apoptosis (Stanzani et al. 2017; Zhou et al. 2020). Activation of JAK2/STAT3 signaling in pancreatic cancer can lead to tumorigenesis, progression, cancer stem cell maintenance, and treatment resistance (Tyagi et al. 2016). CHMP4C affected the proliferation of lung cancer cells by cell cycle pathway in squamous cell carcinoma of the lung (Liu et al. 2021b). In summary, we speculated that CDC45 may be involved in the progression of KIRC through these pathways, however, this remained to be further verified by subsequent experiments.

Subsequently, to further explore the pathogenesis, we screened ten genes that closely interact with CDC45. MCM2, MCM3, MCM4, MCM5, MCM6, MCM7 as key proteins in the initiation of DNA replication played a crucial role in gene stabilization (Neves and Kwok 2017; Wang et al. 2020). Studies had shown that aberrant expression of MCM2-7 caused failure of DNA replication leading to tumorigenesis (Pruitt et al. 2007; Wu et al. 2018). As GINS2, GINS4, which were also involved in the DNA replication process, previous studies had reported that they were abnormally expressed in many tumor tissues including KIRC, affecting tumor development. In hepatocellular carcinoma, up-regulation of GINS2 expression suggested a poorer prognosis and GINS2 may influence the extent of immune cell infiltration and thus the tumor microenvironment and ultimately altered the immune response (Li et al. 2022). However, the mechanisms involved were not yet clear. The study by Jin et al. concluded that GINS4 was associated with immune cell infiltration in esophageal squamous cell carcinoma and may play a role in the immune system (Jin et al. 2022). Taken together, the current state of research described above suggested that CDC45 may play an indispensable role in KIRC through these genes however the exact mechanism remained to be explored.

As far as immunity was concerned, CDC45 showed significant associations with immune cell infiltration and immune checkpoints (*p* < 0.05). As part of immune surveillance, the immune system identifies, kills and removes mutated cells in the body before tumors form. Tumor cells evade immune surveillance by using immune checkpoints to masquerade as normal cells in the body. Blocking the immune checkpoint pathway to prevent tumor cells from masquerading as normal components of the body was an effective way to achieve anti-tumor immunity (Li et al. 2019b). In this paper, we found that CDC45 was associated with immune checkpoints, suggesting that through the regulation of particular immune checkpoint genes, CDC45 may regulate tumor immune patterns. And it provided a new idea for the future immunotherapy of KIRC.

In general, there were some highlights in our study. First, we used the TCGA database to identify the elevated expression of CDC45 in KIRC and further verified its expression by PCR. Second, we further determined the relationships between CDC45 and immunity through single-cell and bulk RNA-sequencing and predicted the response to immunotherapy in KIRC patients. However, there were still some shortcomings in this paper. First of all, we did not experimentally verify CDC45 expression at the protein level. Last but not least, whether the pathways associated with CDC45 in this paper affected the progression of KIRC remained to be verified by further experiments.

## Conclusion

In summary, this paper validated increased CDC45 expression in KIRC by bioinformatics analysis and demonstrated its potential as a prognostic biomarker of KIRC. In addition, CDC45 correlated with the level of immune cell infiltration in KIRC, suggesting that CDC45 may play a significant role in controlling the immune microenvironment of KIRC, but its specific mechanism still needed to be further investigated.

### Supplementary Information

Below is the link to the electronic supplementary material.

Supplementary file1 Figure S1. The annotated cell types in the GSE159115 dataset by UMAP plot. (TIF 1845 KB)

## Data Availability

The RNA-sequencing data and corresponding clinical information were downloaded from the Cancer Genome Atlas (TCGA) database (https://portal.gdc.cancer.gov/). Immunohistochemical results of CDC45 were downloaded from Human Protein Atlas (HPA) online website (http://www.proteinatlas.org/).
