# Supplementary figures and images for: Elevated CDC45 Expression Predicts Poorer Overall Survival Prognoses and Worse Immune Responses for Kidney Renal Clear Cell Carcinoma via Single-Cell and Bulk RNA-Sequencing
Source: Biochem Genet. 2023 Aug 29;62(3):1502–20. doi: 10.1007/s10528-023-10500-y (PMC11186877; doi:10.1007/s10528-023-10500-y)

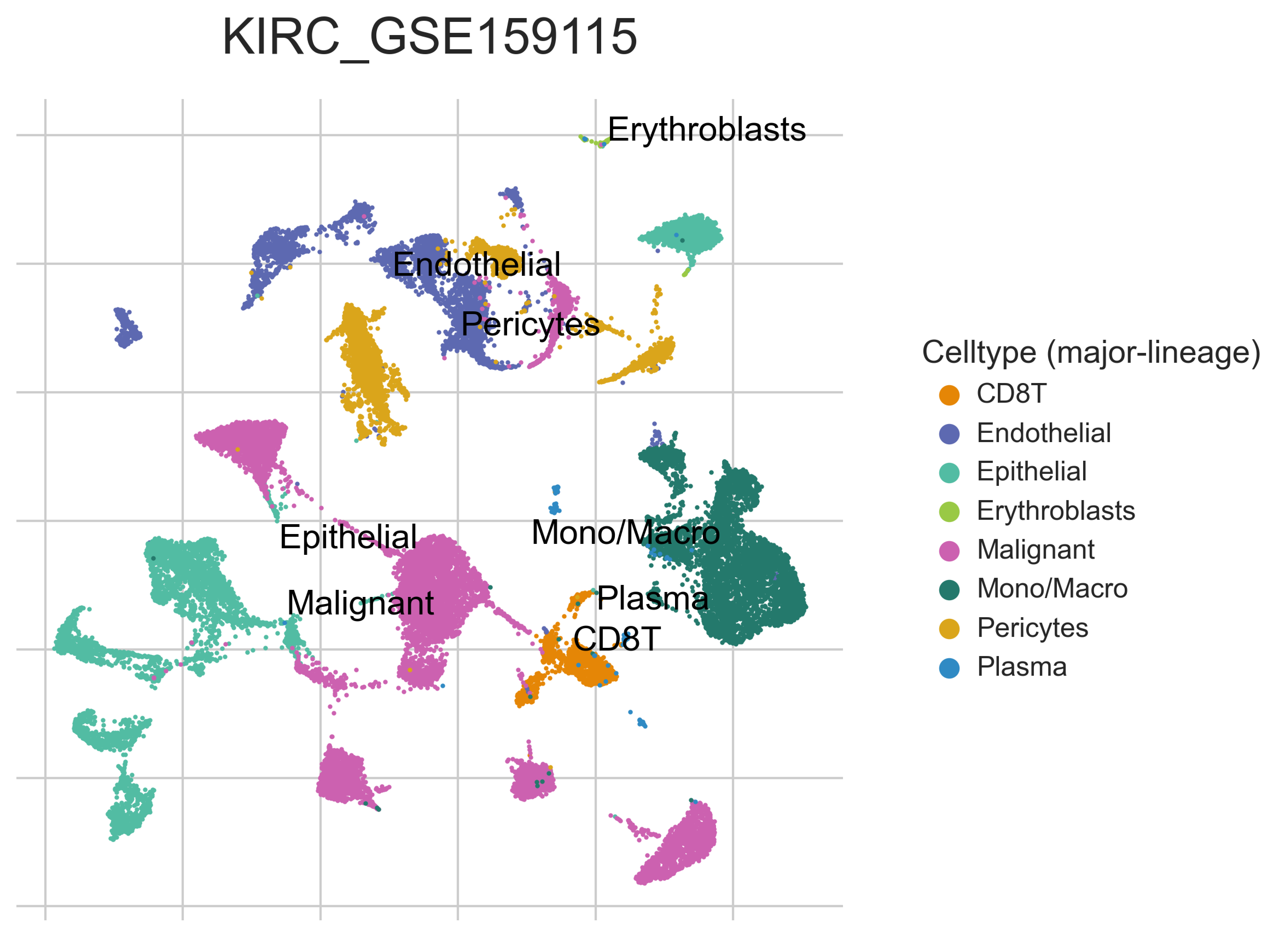

Supplement: Supplementary file 1 — Supplementary file1 Figure S1. The annotated cell types in the GSE159115 dataset by UMAP plot. (TIF 1845 KB) [file 10528_2023_10500_MOESM1_ESM.tif]
